# Retrospective analysis of the prognostic implications of tumor spread through air spaces in lung adenocarcinoma patients treated with surgery

**DOI:** 10.1016/j.esmoop.2022.100568

**Published:** 2022-08-22

**Authors:** L. Gutierrez-Sainz, S. López-Muñoz, P. Cruz-Castellanos, O. Higuera, M.I. Esteban-Rodríguez, I. Losantos-García, J. De Castro-Carpeño

**Affiliations:** 1 Medical Oncology Department, Hospital Universitario La Paz, IdiPAZ, Madrid, Spain; 2 Pathology Department, Hospital Universitario La Paz, Madrid, Spain; 3 Translational Oncology Group, IdiPAZ, Madrid, Spain; 4 Biostatistics Department, Hospital Universitario La Paz, Madrid, Spain; 5 Faculty of Medicine, Universidad Autónoma de Madrid, Madrid, Spain

**Keywords:** lung adenocarcinoma, surgery, tumor spread through air spaces

## Abstract

**Background:**

Tumor spread through air spaces (STAS) in lung adenocarcinoma is a novel mechanism of invasion. STAS has been proposed as an independent predictor of poor prognosis. The aim of this study was to evaluate the correlations between STAS status and other clinicopathologic variables and to assess the prognostic implications of STAS and the distance from the edge of the tumor to the farthest STAS in patients with resected lung adenocarcinoma.

**Material and methods:**

This is a single-institution retrospective observational study. We included all patients with resected lung adenocarcinoma from January 2017 to December 2018 at La Paz University Hospital. The cut-off for the distance from the edge of the tumor to the farthest STAS was 1.5 mm and was assessed by the area under the receiver operating characteristic curve.

**Results:**

A total of 73 patients were included. STAS was found in 52 patients (71.2%). Histological grade 3 (*P* = 0.035) and absence of lepidic pattern (*P* = 0.022) were independently associated with the presence of STAS. The median recurrence-free survival (RFS) was 48.06 months [95% confidence interval (CI) 33.58 months to not reached]. STAS-positive patients had shorter median RFS [39.23 months (95% CI 29.34-49.12 months)] than STAS-negative patients (not reached) (*P* = 0.04). STAS-positive patients with a distance from the edge of the tumor to the farthest STAS ≥1.5 mm had an even shorter median RFS [37.63 months (95% CI 28.14-47.11 months)]. For every 1 mm increase in distance, the risk of mortality increased by 1.26 times (*P* = 0.04).

**Conclusions:**

Histological grade 3 and absence of lepidic pattern were independently associated with the presence of STAS. STAS was associated with a higher risk of recurrence. The distance from the edge of the tumor to the farthest STAS also had an impact on overall survival.

## Highlights

• Lung adenocarcinoma patients with STAS had higher risk of recurrence.
• Patients with STAS and a distance from the edge of the tumor to the farthest STAS ≥1.5 mm had an even shorter RFS.
• The distance from the edge of the tumor to the farthest STAS also had an impact on overall survival.

## Introduction

Lung cancer is the leading cause of cancer-related death worldwide.^1^ Spread through air spaces (STAS) is defined as spread of micropapillary clusters, solid nests or single cancer cells into air spaces in the lung parenchyma beyond the edge of the main tumor.^2^ In 2005, Shiono et al.^3^ reported that the presence of aerogenous spreads with floating cancer cell clusters was a prognostic factor significantly related to local recurrence in patients with pulmonary metastasis from colorectal cancer. In 2013, Onozato et al*.*^4^ described the existence of tumor islands located at the periphery of the lesion which were separated from the main tumor by at least a few alveoli in patients with lung adenocarcinoma treated with surgery. The name STAS, however, was coined by Kadota et al.^5^ in 2015. In the same year, the World Health Organization (WHO) considered STAS as a novel mechanism of invasion.^1^^,^^6^

The incidence of STAS in lung adenocarcinoma ranges from 15% to 73% in related literature.^7^, ^8^, ^9^, ^10^, ^11^, ^12^, ^13^ Since 2015, a large variety of studies have described the association of STAS and clinicopathologic features.^7^, ^8^, ^9^, ^10^, ^11^, ^12^, ^13^, ^14^ The presence of STAS was associated with higher tumor stage, nodal involvement, micropapillary and solid growth patterns, absence of lepidic component, lymphovascular and perineural invasion and moderate/poorly differentiated tumors.^7^, ^8^, ^9^, ^10^, ^11^, ^12^, ^13^ In addition, a high density of tumor-associated macrophage infiltration was related with an increased STAS rate.^15^ In terms of molecular alteration, the conclusions of recent studies are controversial. Several studies have found that STAS was associated with epidermal growth factor receptor (EGFR) wild type,^9^^,^^12^^,^^16^ whereas Tian et al.^9^ reported that EGFR was one of the most frequent alterations found in STAS-positive patients. Regarding anaplastic lymphoma kinase (ALK) mutations, some studies have found a high association between STAS and ALK mutations.^9^^,^^11^^,^^16^ Other molecular alterations described in patients with STAS were tumor protein p53, Kirsten rat sarcoma viral oncogene (KRAS) and ROS proto-oncogene 1 (ROS1).^9^^,^^16^ To date, STAS has not been significantly associated with programmed death-ligand 1 (PD-L1) expression.^17^

Lymphovascular invasion, pleural invasion and infiltration of the stroma are well known patterns of invasion in lung adenocarcinoma and are related to poor prognosis. Currently, several studies have focused on the relationship between STAS and prognosis.^14^^,^^16^^,^^18^, ^19^, ^20^, ^21^, ^22^, ^23^, ^24^, ^25^, ^26^, ^27^, ^28^, ^29^ STAS has been associated with shorter recurrence-free survival (RFS) and overall survival (OS) in lung adenocarcinoma patients treated with surgery; suggesting that STAS could be an independent predictor of recurrence.^14^^,^^16^^,^^18^, ^19^, ^20^, ^21^, ^22^, ^23^, ^24^, ^25^, ^26^, ^27^, ^28^, ^29^ According to the pathological stage, a few studies specifically reported that STAS was associated with shorter RFS in stage I,^18^^,^^20^, ^21^, ^22^^,^^24^, ^25^, ^26^, ^27^, ^28^, ^29^ whereas other studies focused on stage II and III.^18^^,^^23^ These results are supported by two meta-analyses.^30^^,^^31^ Regarding the extension of STAS, Warth et al.^32^ and Dai et al*.*^33^ described limited and extensive STAS with the distance of three alveoli as the cut-off, without obtaining differences in survival between both groups. Recently, Han et al.^34^ graded the extent of STAS according to the distance from the edge of the tumor to the farthest STAS, obtaining two groups of patients based on whether the presence of STAS was closer to or further than 2.5 mm. The conclusion of their study was that there were significant differences in RFS and OS according to the extent of STAS, specifically patients with STAS further than 2.5 mm had shorter survival. Additionally, Uruga et al.^35^ classified STAS into low STAS (one to four single cells or clusters) and high STAS (five or more single cells or clusters) and found that patients with high STAS had shorter RFS and OS than patients with low STAS.

In lung adenocarcinoma, there are different surgical procedures such as sublobar resection, lobectomy and pneumonectomy depending on the tumor features and the patient’s overall condition.^36^ There is no consensus whether sublobar resection increases the risk of locoregional recurrence compared with lobectomy in patients with STAS.^20^^,^^36^, ^37^, ^38^, ^39^ A few studies suggested that sublobar resection was associated with a higher risk of recurrence in patients with stage IA and presence of STAS.^20^^,^^36^^,^^37^ Kagimoto et al.^40^ described that prognosis after sublobar resection, however, was comparable with that of lobectomy in lung adenocarcinoma with STAS without increasing locoregional recurrence. Regarding adjuvant treatment, Chen et al.^29^ found that adjuvant chemotherapy improved outcomes in STAS-positive patients with stage IA who underwent sublobar resection.

The aim of this study was to assess the correlation between STAS status and other clinicopathologic variables. Furthermore, we also expected to explore whether STAS presence and the distance from the edge of the tumor to the farthest STAS were reliable prognostic factors of survival in patients with lung adenocarcinoma.

## material and methods

### Patients and study design

This is a single-institution retrospective observational study. We included all patients with resected lung adenocarcinoma from January 2017 to December 2018 at La Paz University Hospital, Madrid (Spain). Patients were aged ≥18 years. The diagnosis of lung adenocarcinoma was confirmed histologically. Data regarding clinical and demographic characteristics, type of surgery and pathological features were obtained from the medical records of each patient. Staging was carried out in accordance with the standards of the American Joint Committee on Cancer, 8th Edition. Histological grading was measured using the grading system developed by The International Association for the Study of Lung Cancer (IASLC)^41^ and has recently been incorporated into the 2021 WHO Classification of Thoracic Tumors.^42^ Post-operative follow-up consisted of a contrast-enhanced computed tomographic (CT) scan of the chest, abdomen and pelvis every 3 months for the first 2 years after resection, then every 6 months for the next 3 years and annually thereafter. In addition, a contrast-enhanced brain magnetic resonance imaging (MRI) or a contrast-enhanced brain CT scan was carried out if clinically indicated.

This study was approved by the Ethics Committee of the La Paz University Hospital (code HULP: PI-4843), and was conducted in accordance with the ethical standards of the Helsinki Declaration by the World Medical Association.

### Statistical analysis

**Figure 1:**
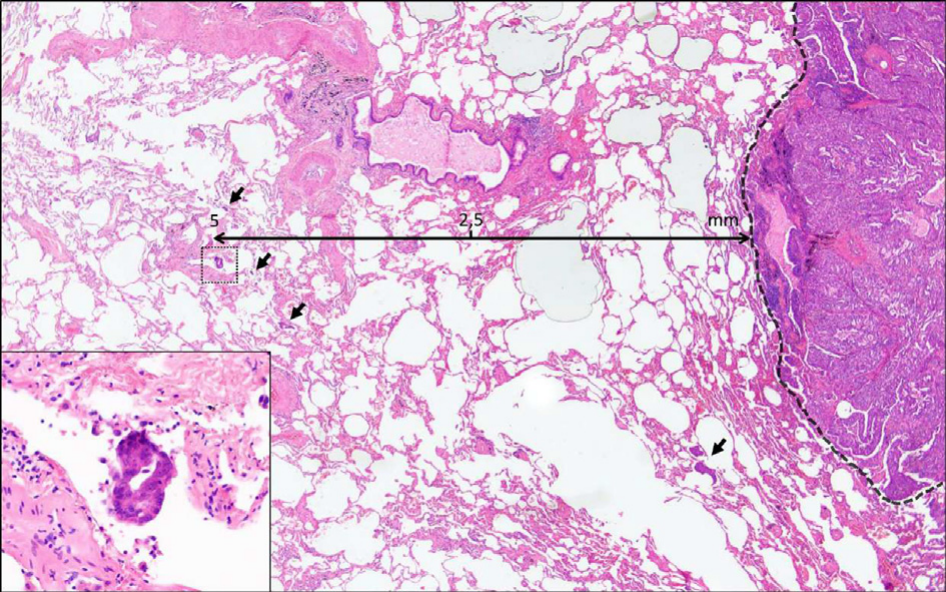
Complex glandular pattern adenocarcinoma of the lung with STAS. Several tumor clusters were identified beyond the edge of the main tumor (black arrows). Zoomed in (×40 magnification) on a micropapillary cluster within air spaces located 5 mm away from the edge of the tumor. STAS, spread through air spaces.

Median value (interquartile range) and frequency (percentage) were provided for the description of continuous and categorical variables, respectively. We used descriptive statistics to calculate the incidence of STAS. The distance from the edge of the tumor to the farthest STAS was also measured (Figure 1). The cut-off for this distance was assessed by the area under the receiver operating characteristic (ROC) curve.

Comparisons of categorical data between patients with and without STAS were carried out with the chi-square or Fisher’s exact test. Variables that achieved statistical significance in the univariate analysis and other variables considered of interest were included in the multivariate analysis using the logistic regression model. The logistic regression model was carried out to estimate odds ratios (ORs) and 95% confidence intervals (CIs) for clinicopathologic variables associated with STAS status.

RFS was calculated from the date of diagnosis until first recurrence (locoregional or distant metastasis) or death due to any cause. OS was calculated from the date of diagnosis to the date of death from any cause. RFS and OS were compared among patients with and without STAS. Survival was estimated using the Kaplan–Meier method and described using median with 95% CI. A Cox regression was carried out to estimate the hazard ratios (HRs) and the 95% CIs. All the tests were two-sided, and *P* values < 0.05 were considered statistically significant. All statistical analyses were carried out using SPSS v.25.

## Results

### Incidence of STAS and clinicopathologic characteristics of the patients

A total of 73 patients were included. STAS was found in 52 patients (71.2%). Baseline clinical and demographic characteristics, type of surgery, pathological features and molecular alterations of the patients with and without STAS are summarized in Table 1*.*

**Table 1:** Baseline characteristics of patients with and without STAS

Variables	All patients	STAS (+)	STAS (−)	*P* value
	*N* = 73	*N* = 52	*N* = 21	
Age (years), *n* (%):				
≥70	35 (47.9)	28 (80.0)	7 (20.0)	
<70	38 (52.1)	24 (63.2)	14 (36.8)	0.112
Sex, *n* (%):				
Male	44 (60.3)	31 (70.5)	13 (29.5)	
Female	29 (39.7)	21 (72.4)	8 (27.6)	0.856
Smoking status, *n* (%):				
Smoker or former smoker	60 (82.2)	42 (70.0)	18 (30.0)	
Never smoker	13 (17.8)	10 (76.9)	3 (23.1)	0.745
Type of surgery, *n* (%):				
Sublobar resection	14 (19.2)	11 (78.6)	3 (21.4)	
Lobectomy	59 (80.8)	41 (69.5)	18 (30.5)	0.744
Pathological stage, *n* (%):				
IA	37 (50.8)	22 (59.5)	15 (40.5)	
IB	15 (20.5)	12 (80.0)	3 (18.8)	
IIA	3 (4.1)	2 (66.7)	1 (33.3)	
IIB	10 (13.7)	10 (100)	0 (0.0)	
IIIA	6 (8.2)	4 (66.7)	2 (33.3)	
IIIB	2 (2.7)	2 (100)	0 (0.0)	a
Pathological stage (grouped), *n* (%):				
I	52 (71.2)	34 (65.4)	18 (34.6)	
II and III	21 (28.8)	18 (85.7)	3 (14.3)	0.082
Nodal stage, *n* (%):				
N0	62 (84.9)	42 (67.7)	20 (32.3)	
N1	8 (11.0)	7 (87.5)	1 (12.5)	
N2	3 (4.1)	3 (100.0)	0 (0.0)	0.271
Positive surgical margins, *n* (%):				
Yes	1 (1.4)	1 (100)	0 (0.0)	
No	72 (98.6)	51 (70.8)	21 (29.2)	a
Histological grading, *n* (%):				
Grade 1	3 (4.1)	0 (0.0)	3 (100)	
Grade 2	36 (49.3)	22 (61.1)	14 (38.9)	
Grade 3	34 (46.6)	30 (88.2)	4 (11.8)	a
Histological grading (grouped), *n* (%):				
Grade 1 or 2	39 (53.4)	22 (56.4)	17 (43.6)	
Grade 3	34 (46.6)	30 (88.2)	4 (11.8)	**0.004**
Lymphovascular invasion, *n* (%):				
Yes	15 (20.5)	13 (86.7)	2 (13.3)	
No	58 (79.5)	39 (67.2)	19 (32.8)	0.204
Perineural invasion, *n* (%):				
Yes	3 (4.1)	3 (100)	0 (0.0)	
No	70 (95.9)	49 (70.0)	21 (30.0)	a
Lepidic pattern, *n* (%):				
Yes	26 (35.6)	13 (50.0)	13 (50.0)	
No	47 (64.4)	39 (83.0)	8 (17.0)	**0.003**
Acinar pattern, *n* (%):				
Yes	58 (79.5)	40 (69.0)	18 (31.0)	
No	15 (20.5)	12 (80.0)	3 (20.0)	0.400
Papillary pattern, *n* (%):				
Yes	20 (27.4)	14 (70.0)	6 (30.0)	
No	53 (72.6)	38 (71.7)	15 (28.3)	0.886
Solid pattern, *n* (%):				
Yes	26 (35.6)	24 (92.3)	2 (7.7)	
No	47 (64.4)	28 (59.6)	19 (40.4)	**0.003**
Micropapillary pattern, *n* (%):				
Yes	20 (27.4)	15 (75.0)	5 (25.0)	
No	53 (72.6)	37 (69.8)	16 (30.2)	0.662
EGFR, *n* (%):				
Positive	8 (11.0)	5 (62.5)	3 (37.5)	
Negative	65 (89.0)	47 (72.3)	18 (27.7)	0.682
ALK, *n* (%):				
Positive	3 (4.1)	3 (100)	0 (0.0)	
Negative	70 (95.9)	49 (70.0)	21 (30.0)	a
ROS1, *n* (%):				
Positive	0 (0.0)	0 (0.0)	0 (0.0)	
Negative	43 (58.9)	28 (65.1)	15 (34.9)	
Unknown	30 (41.1)	24 (80.0)	6 (20.0)	a
PD-L1 status, *n* (%):				
<1%	34 (46.6)	23 (67.6)	11 (32.4)	
1-49%	20 (27.4)	14 (70.0)	6 (30.6)	
≥50%	19 (26.0)	15 (78.9)	4 (21.1)	0.677

Values in bold are statistically significant.

**Smoker:** An adult who has smoked at least 100 cigarettes in his or her lifetime, and who now smokes every day. Previously called a “regular smoker”.

**Former smoker:** An adult who has smoked at least 100 cigarettes in his or her lifetime but who had quit smoking at the time of interview.

**Never smoker:** An adult who has never smoked, or who has smoked less than 100 cigarettes in his or her lifetime.

ALK, anaplastic lymphoma kinase; EGFR, epidermal growth factor receptor; PD-L1, programmed death-ligand 1; ROS1, ROS proto-oncogene 1; STAS, spread through air spaces.

a A *P* value could not be obtained due to lack of cases. Therefore, we grouped some of these variables to obtain a *P* value.

The majority (*n* = 44, 60.3%) were males with a median age of 68 years (range 42-85 years). A total of 60 patients (82.2%) were smokers or former smokers. Most patients (*n* = 52, 71.2%) had pathological stage I, 13 patients (17.8%) had pathological stage II and 8 patients had pathological stage III (10.9%). The minority (*n* = 11, 15.1%) had confirmed nodal involvement. Only one patient (1.4%) had positive surgical margins. Regarding histological grading, approximately half of the patients had grade 3 (*n* = 34, 46.6%) and half of the patients had grade 2 (*n*= 36, 49.3%) with a few patients with grade 1 (*n* = 3, 4.1%). The existence of lymphovascular and perineural invasion occurred in 20.5% and 4.1% of the patients, respectively. The most frequent histological pattern was acinar (*n* = 58, 80.6%). Around one-third of the patients (*n* = 26, 35.6%) had lepidic pattern, another one-third of patients (*n* = 26, 35.6%) had solid pattern, 20 patients (27.4%) had papillary pattern and 20 patients (27.4%) had micropapillary pattern. In terms of molecular alteration, eight patients (11%) had EGFR mutations, three patients (4.1%) had ALK mutations and no patient had ROS1 mutations. Almost half of the patients had PD-L1 expression <1% (*n* = 34, 46.6%), 20 patients (27.4%) had PD-L1 expression between 1% and 49% and 19 patients (26%) had PD-L1 expression ≥50%. Regarding perioperative treatment, 17 patients (23.3%) received adjuvant chemotherapy and 2 (2.7%) patients received neoadjuvant chemotherapy.

The presence of STAS was more frequently observed in patients with pathological stage II or III (all patients with stage IIB and IIIB and two-thirds of patients with stage IIA and IIIA), in patients with nodal involvement (87.5% and 100% for N1 and N2, respectively, versus 67.7%), in patients with histological grade 3 (88.2% versus 56.4%), in patients with lymphovascular (86.7% versus 67.2%) and perineural invasion (100% versus 70%), in patients with absence of lepidic pattern (83% versus 50%), in patients with solid (92.3% versus 59.6%) and micropapillary patterns (75% versus 69.8%), in patients with PD-L1 expression ≥50% (78.9% versus 70 and 67.6%), in patients with EGFR wild type (72.3% versus 62.5%) and in patients with ALK mutations (100% versus 70%).

Sublobar resection (all of which were segmentectomies) was carried out in 14 patients (19.2%) and lobectomy in 59 patients (80.8%). Interestingly, STAS was slightly more prevalent in sublobar resections (78.6% versus 69.5%, respectively) (Table 1). Regarding the pathological stage of patients who underwent sublobar resection, 12 patients had stage I and two patients had stage IIIA. These two patients with pathological stage IIIA were due to having a separated tumor nodule in a different ipsilateral lobe. None of the patients who underwent sublobar resection had lymph node involvement.

### Correlation of STAS with other clinicopathologic variables

Among the clinicopathological characteristics, histological grade 3 (*P* = 0.004), solid pattern (*P* = 0.003) and absence of lepidic component (*P* = 0.003) were significantly associated with the presence of STAS in the univariate analysis (Table 1). Owing to solid pattern and histological grade 3 which were associated with each other (*P* < 0.001), we did not enter solid pattern in the final multivariate model. We also included other variables considered of interest such as age and sex in the multivariate analysis.

In the multivariate analysis, histological grade 3 [OR 4.10 (1.10-15.25), *P* = 0.035] and absence of lepidic pattern [OR 0.25 (0.07-0.81), *P* = 0.022] were independently associated with the presence of STAS (Table 2).

**Table 2:** Multivariate binary logistic regression analysis with STAS as the dependent variable

Variables	Multivariate analysis	
	OR (95% CI)	*P* value
Age (years):		
≥70 versus <70	2.32 (0.70**-**7.71)	0.169
Sex:		
Male versus female	0.75 (0.22**-**2.49)	0.638
Lepidic pattern:		
Yes versus no	**0.25 (0.07-0.81)**	**0.022**
Histological grading (grouped):		
Grade 3 versus grade 1 or 2	**4.10 (1.10-15.25)**	**0.035**

Values in bold are statistically significant.

CI, confident interval; OR, odds ratio; STAS, spread through air spaces.

### Prognostic significance of STAS

**Figure 2:**
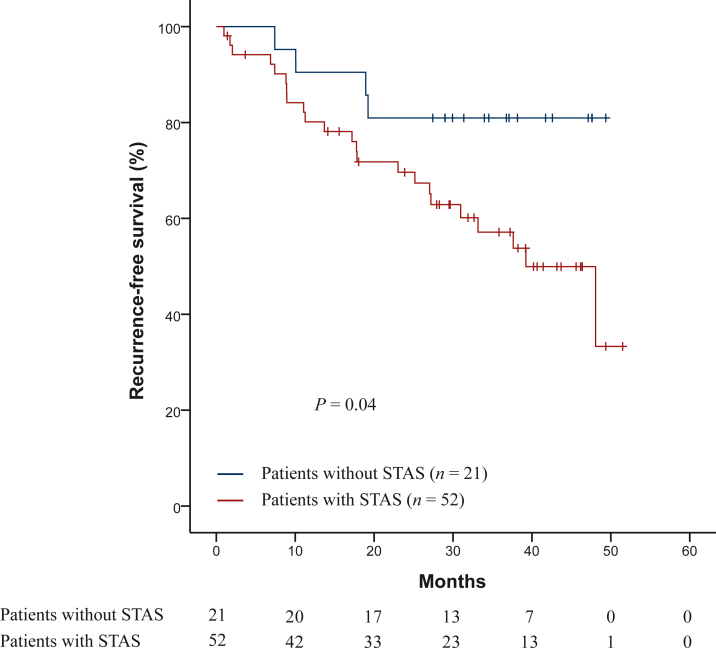
**Kaplan–Meier curves for recurrence-free survival in patients with and without STAS.** STAS, spread through air spaces.

A total of 27 patients (37%) had confirmed recurrence of the disease, of whom 21 patients underwent lobectomy and 7 patients underwent sublobar resection. Regarding the pathological stage of patients who had confirmed recurrence of the disease, 12 patients had stage I (23% of the total of patients with stage I), 7 patients stage II (53.8% of the total of patients with stage II) and 8 patients stage III (100% of the total of patients with stage III). The most common site of recurrence was the lung (*n* = 14, 51.9%), followed by the brain (*n* = 6, 22.2%), the pleura (*n* = 3, 11.1%), the lymph nodes (*n* = 3, 11.1%) and bone (*n* = 1, 1.4%). The median RFS was 48.06 months (95% CI 33.58 months to not reached). STAS-positive patients had shorter median RFS (39.23 months, 95% CI 29.34-49.12 months) than STAS-negative patients (not reached), with statistically significant differences (*P* = 0.04) (Figure 2). The risk of recurrence was 2.8 times higher in patients with STAS (HR: 2.8, 95% CI 0.97-8.13), but without statistically significant differences (*P* = 0.05). Specifically in patients who underwent lobectomy, STAS-positive patients also had shorter median RFS than STAS-negative patients (median 39.23 months versus not reached), but without statistically significant differences (*P* = 0.11). Taking into account the distance from the edge of the tumor to the farthest STAS in patients who underwent lobectomy, for every 1 mm increase in distance, the risk of recurrence increased by 1.17 times [HR 1.17 (95% CI 1.01-1.36 times)] with statistically significant differences (*P* = 0.03).

**Figure 3:**
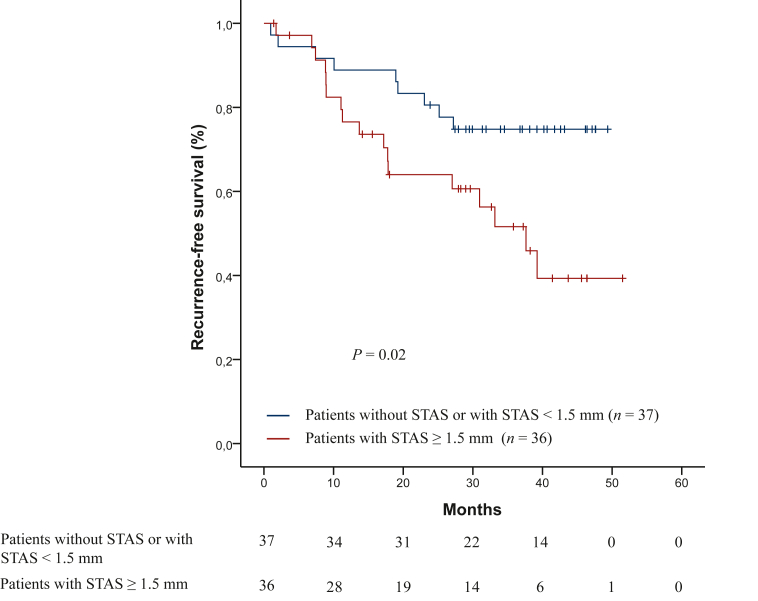
Kaplan–Meier curves for recurrence-free survival in patients with STAS at a distance from the edge of the tumor to the farthest STAS ≥1.5 mm and without STAS or STAS at a distance from the edge of the tumor to the farthest STAS shorter than 1.5 mm. STAS, spread through air spaces.

The cut-off for the distance from the edge of the tumor to the farthest STAS was 1.50 mm. A total of 36 patients (49.3%) had STAS at a distance from the edge of the tumor to the farthest STAS ≥1.5 mm, of whom 6 patients underwent sublobar resection and 30 patients underwent lobectomy. STAS-positive patients with a distance from the edge of the tumor to the farthest STAS ≥1.5 mm had shorter median RFS [37.63 months (95% CI 28.14-47.11 months)] than STAS-negative patients or STAS-positive patients with a distance from the edge of the tumor to the farthest STAS shorter than 1.5 mm (not reached), with statistically significant differences (*P* = 0.02) (Figure 3). The risk of recurrence was 2.4 times higher in patients with STAS and a distance from the edge of the tumor to the farthest STAS ≥1.5 mm (HR: 2.4, 95% CI 1.09-5.56), with statistically significant differences (*P* = 0.02). According to the type of surgery, patients with STAS who underwent a sublobar resection had shorter RFS (48.06 months) than patients with STAS who underwent a lobectomy (39.23 months), but without statistically significant differences (*P* = 0.83). Specifically in patients who underwent lobectomy, STAS-positive patients with a distance from the edge of the tumor to the farthest STAS ≥1.5 mm also had shorter median RFS than STAS-negative patients or STAS-positive patients with a distance from the edge of the tumor to the farthest STAS <1.5 mm (median 37.63 months versus not reached), with statistically significant differences (*P* = 0.02). The risk of recurrence was 2.6 times higher in patients with STAS and a distance from the edge of the tumor to the farthest STAS ≥1.5 mm (HR: 2.6, 95% CI 1.07-6.65), with statistically significant differences (*P* = 0.02).

A total of 15 patients (20.5%) had died, of whom 1 patient underwent sublobar resection and 14 patients underwent lobectomy. The median OS was not reached. STAS-positive patients appeared to have shorter OS than STAS-negative patients, however medians were not reached. The distance from the edge of the tumor to the farthest STAS had an impact on OS. For every 1 mm increase in distance, the risk of death increased by 1.26 times [HR 1.26 (95% CI 1.00-1.59)] with statistically significant differences (*P* = 0.04). Specifically in patients who underwent lobectomy, STAS-positive patients also appeared to have shorter OS, but medians were not reached either. In addition, the distance from the edge of the tumor to the farthest STAS also had an impact on OS in these patients. For every 1 mm increase in distance, the risk of death also increased by 1.26 times [HR 1.26 (95% CI 1.00-1.59)] with statistically significant differences (*P* = 0.04).

## Discussion

In this study, we analyzed the clinical implications and the prognosis of the presence of STAS in lung adenocarcinoma patients treated with surgery. We observed that histological grade 3 and absence of lepidic pattern were independently associated with the presence of STAS. We also found that STAS-positive patients had a higher risk of recurrence than patients without STAS. In addition, STAS-positive patients with a distance from the edge of the tumor to the farthest STAS ≥1 mm had an even shorter median RFS. Regarding OS, we noted that STAS-positive patients appeared to have shorter OS but medians were not reached and the greater the distance from the edge of the tumor to the farthest STAS, the greater the risk of death.

The incidence of STAS in our study was 71.2%, which was higher compared with the incidence observed by other authors (27.9%,^7^ 46%,^8^ 50.6%,^11^ 26.8%^12^ and 32.4%^13^). In contrast, Toyokawa et al*.*^14^ reported that STAS was found in 73% of patients and suggested that STAS may be more frequently observed in more advanced cases of lung adenocarcinoma. The reason for our high incidence of STAS could be explained by the fact that we only included patients with adenocarcinoma, and apart from stage I we also included stage II and III patients. In recent years, several studies have analyzed the correlation between STAS and other clinicopathologic features.^7^, ^8^, ^9^, ^10^, ^11^, ^12^, ^13^ We found that histological grade 3 and absence of lepidic pattern were independently associated with the presence of STAS. We also found that solid pattern was associated with the presence of STAS in the univariate analysis. Solid pattern, however, could not be entered into the multivariate model. Lee et al.^11^ and Hu et al.^12^ also identified a correlation between poorly differentiated subtypes and the presence of STAS. In other studies as well,^8^^,^^11^, ^12^, ^13^ STAS occurred less frequently in lepidic-predominant adenocarcinomas. Regarding solid pattern, Xie et al.^8^ and Cao et al.^13^ also reported that solid pattern was significantly associated with the presence of STAS. Other clinicopathologic characteristics associated with STAS in the literature were higher tumor stage, nodal involvement, micropapillary pattern, lymphovascular invasion and perineural invasion.^7^, ^8^, ^9^, ^10^, ^11^, ^12^, ^13^ We probably did not find statistically significant differences between these variables and the presence of STAS due to the short follow-up time and the small sample size.

We also evaluated the association between STAS and molecular mutations. In our study, the presence of STAS was more frequently observed in patients with ALK mutations and EGFR wild type, but without statistically significant differences. In the literature, the results of previous studies are contradictory. Regarding ALK mutations, Tian et al.,^9^ Lee et al.^11^ and Jia et al.^16^ also described that there was a significant association between STAS and ALK rearrangements. Regarding EGFR mutations, however, Lee et al.^11^ found a relationship between STAS and wild-type EGFR. Tian et al.,^9^ however, reported that STAS was more frequent in patients with positive EGFR mutations.^12^ We did not find any association between STAS and PD-L1 expression. Toyokawa et al.^17^ documented that STAS was not significantly associated with PD-L1 expression. The relationship between STAS and molecular alterations as well as PD-L1 status should be explored further in future studies.

We noted that STAS was a significant risk factor for recurrence in lung adenocarcinoma patients treated with surgery. STAS-positive patients had shorter median RFS than STAS-negative patients with statistically significant differences and the risk of recurrence was 2.8 times higher, but without statistically significant differences. To date, several studies have also found an association between STAS and shorter RFS in lung adenocarcinoma patients treated with surgery independent of the pathological stage.^14^^,^^16^^,^^18^, ^19^, ^20^, ^21^, ^22^, ^23^, ^24^, ^25^, ^26^, ^27^, ^28^, ^29^ In addition, we measured the distance from the edge of the tumor to the farthest STAS and we assessed the cut-off for this distance by the area under the ROC curve, which was 1.5 mm. We found that STAS-positive patients with a distance from the edge of the tumor to the farthest STAS ≥1.5 mm had an even shorter median RFS and their risk of recurrence was 2.4 times higher, with statistically significant differences. Warth et al*.*^32^ initially described limited and extensive STAS with the distance of three alveoli as the cut-off. They did not, however, obtain differences in survival between both groups. Dai et al.^33^ used the same cut-off and neither obtained differences. Recently, Han et al.^34^ graded the extent of STAS differentiating two groups of patients based on whether all tumor clusters were closer to or further than 2.5 mm from the edge of the tumor and showed that patients with the presence of STAS further than 2.5 mm had shorter survival. Uruga et al*.*,^35^ however, classified STAS into low and high STAS depending on the number of single cells or clusters of STAS that patients had and found that patients with high STAS had shorter survival. Further studies are needed to determine the standard method of grading the extension of STAS. In this respect, it is also important to train pathologists in the identification of STAS.

Regarding the type of surgery we found that STAS was slightly more prevalent in sublobar resections, but without statistically significant differences, probably due to the small sample size. Furthermore, patients with STAS who underwent a sublobar resection had shorter RFS than patients who underwent a lobectomy, although we did not reach significant differences. In the subgroup of patients who underwent lobectomy, however, STAS-positive patients also had shorter median RFS than STAS-negative patients, but without statistically significant differences. In the literature, it is still controversial whether sublobar resection increases the risk of locoregional recurrence compared with lobectomy in patients with STAS.^20^^,^^37^, ^38^, ^39^ A few studies agreed that sublobar resection was associated with higher risk of recurrence in patients with STAS,^20^^,^^36^^,^^37^ whereas Kagimoto et al.^40^ disagreed. Taking this into account, it is reasonable to suggest that STAS-positive patients who undergo sublobar resection may potentially benefit from a completion lobectomy or adjuvant therapy to decrease the risk of recurrence. On this matter, Chen et al.^29^ suggested that adjuvant chemotherapy might be considered for STAS-positive patients with stage IA. Further studies are needed to discuss whether these patients need a completion lobectomy or to receive post-operative adjuvant therapy.

Some limitations of our study should be addressed. First, its retrospective nature and the involvement of only one institution. Second, we only included three molecular alterations (EGFR, ALK and ROS1). Therefore, further studies on the relationship between STAS and other molecular alterations are needed. Third, the follow-up was not enough to obtain results of OS, because we only included patients since 2017. Despite these limitations, this study provides relevant information about the incidence of STAS and the relationship between STAS and other clinicopathological characteristics and molecular alterations. In addition, our study reaffirms the poor prognosis related to the presence of STAS in adenocarcinoma patients treated with surgery and provides relevant information on the importance of taking into account the distance from the edge of the tumor to the furthest STAS, since the greater the distance, the worse the survival.

In conclusion, histological grade 3 and absence of lepidic pattern were independently associated with the presence of STAS. In addition, the presence of STAS was associated with a higher risk of recurrence in patients with lung adenocarcinoma treated with surgery. In particular, STAS-positive patients with a distance from the edge of the tumor to the farthest STAS ≥1.5 mm had an even shorter median RFS. Furthermore, the distance from the edge of the tumor to the farthest STAS also had an impact on survival. Further prospective studies including data from multiple centers are needed to derive definitive conclusions.
